# Immunological consequences of past transfusions in kidney transplant candidates: A focus on anti-HLA antibody formation

**DOI:** 10.1016/j.htct.2025.103990

**Published:** 2025-10-14

**Authors:** Tamara Perić, Svetlana Vojvodić

**Affiliations:** a PHI Hospital “Sveti Vračevi” Bijeljina, Bosnia and Herzegovina; b University of Novi Sad, Faculty of Medicine, Novi Sad, Serbia; c University of Novi Sad, Faculty of Medicine, Novi Sad, Department for Blood Transfusion of Vojvodina, Serbia

**Keywords:** Blood transfusion, Anti-HLA antibodies, Sensitization, Panel reactive antibodies test, Alloimmunization

## Abstract

**Introduction:**

Blood transfusions are crucial for saving lives but can affect the recipient's immune system. A significant concern is the development of anti-human leukocyte antigen (HLA) antibodies, which can influence organ transplantation outcomes. The presence of these antibodies increases the risk of transplant rejection. The aim of this study was to evaluate how blood component characteristics (leukodepletion, type, number, volume) and timing from the last transfusion to anti-HLA antibody detection affect sensitization in kidney transplant candidates.

**Materials and Methods:**

This retrospective study analyzed 115 candidates on the cadaveric kidney transplant list from South Bačka and Novi Sad, Serbia. Among them, 69 received blood transfusions, classified as either leukodepleted or containing leukocytes (WBCs), for sensitization control. Anti-HLA antibodies were detected using Complement-Dependent Cytotoxicity, Enzyme-Linked Immunosorbent Assay, and Luminex technology. This study evaluated demographic data, transfusion history, and sensitization. Statistical analysis focused on the relationship between sensitization and blood component variables.

**Results:**

In this study, 53.7 % were sensitized. The number of blood components received (p-value = 0.437), blood unit (p-value = 0.6809), and blood volume (p-value = 0.5857) were not significantly associated with sensitization rates. The use of leukodepleted blood components (p-value = 0.0057), as well as blood components containing WBCs (p-value = 0.030) is associated with a higher sensitization. Sensitization was detected in 67.57 % of cases more than 12 months after transfusion (p-value = 0.046). A significant difference in sensitization was shown when packed red blood cells were used (89.19 % versus 68.75 %; p-value = 0.006).

**Conclusions:**

Sensitization was higher with blood components containing WBCs and packed RBCs. The longer time after transfusion, the more often sensitization is detected.

## Introduction

According to a 2023 report,^1^ the overall kidney transplant rate across 40 European countries increased by 1.9 % from 2010 to 2018. Notably, this rate remains higher in Western Europe compared to Eastern Europe. Serbia, however, experienced a 10.8 % decrease in the kidney transplant rate during this period and a significant 46.0 % reduction in live donor kidney transplant rate from 2016 to 2018, positioning it among the top six and top nine countries in these respective categories. Kidney transplantation has a long history in Serbia, with the first procedures conducted in 1970 in Ljubljana and then in 1973 in Belgrade. Over the past 50 years, fewer than 1500 transplants have been performed, with over 70 % being related transplants.^2^ From 2010 to 2021, Serbia carried out 460 kidney transplants, while 725 additional patients were added to the waiting list in 2022.

Kidney transplant is widely recognized as the preferred treatment for end-stage chronic kidney disease (CKD), offering enhanced survival and quality of life. Despite its advantages, kidney transplant is not without significant risks and challenges, including anemia, a common comorbidity in terminal-stage CKD patients. Over the last two decades, management of CKD-related anemia has advanced significantly. Historically, blood transfusions were the primary treatment, but they come with complications such as infections, iron overload, fluid imbalance, and adverse reactions to transfusions.^3^ Additionally, repeated transfusions increase the risk of alloimmunization, complicating outcomes for patients awaiting renal transplantation.^4^ The introduction of recombinant erythropoietin in the late 1980s, followed by erythropoiesis-stimulating agents (ESAs), revolutionized anemia treatment.^5^ These therapies not only reduced the need for transfusions but also improved survival rates, quality of life, cardiac function, and decreased hospital admissions. Importantly, they helped lower the percentage of transplant candidates with high panel reactive antibodies (PRA) levels on the waiting list.^6^

Sensitization refers to the presence of antibodies in a potential transplant recipient's serum, typically against HLA class I or class II antigens, and occasionally against non-HLA antigens. A key tool used to quantify immune sensitization is the PRA test, which estimates the percentage of the general population to which a patient has preformed anti-HLA antibodies. Higher PRA values indicate a greater degree of sensitization and are associated with longer waiting times, increased risk of graft rejection, and poorer transplant outcomes.^7^^,^^8^ Sensitization can occur due to a variety of causes, including blood transfusions, prior transplants, pregnancy, and ventricular assist devices, or sensitization may occasionally arise spontaneously.^9^ Among these factors, blood transfusions are a major contributor, responsible for approximately 20–33 % of sensitization events, especially in patients who did not receive leukoreduced blood components.^10^

Despite red blood cells (RBCs) expressing low levels of HLA class I molecules, their sheer number results in a comparable HLA load to residual WBCs in leukocyte-depleted blood.^11^ Previous research highlights the link between RBC transfusions and HLA sensitization, leading to prolonged waiting times for transplantation and reduced graft survival.^12^ Studies, such as one by Aston et al. in 2014,^13^ found no significant benefit from additional washing of red cells in reducing HLA sensitization risk. Prevention strategies focus on minimizing transfusions by optimizing iron stores, judicious use of erythropoietin, and selecting transplants with the best possible HLA match.

PRA levels are not only diagnostic but also prognostic: high PRA values prior to transplantation correlate with an increased risk of graft rejection and delayed graft function.^13^^,^^8^ For instance, each 1 % increase in PRA above 20 % has been linked to a 5 % rise in rejection risk.^14^

Preformed anti-HLA antibodies pose a significant risk to transplant success, increasing the likelihood of antibody-mediated rejection and graft loss. Allosensitization from blood transfusions exacerbates these risks, limiting graft availability, prolonging waiting times, and shortening graft survival.^15^ Minimizing blood transfusions in CKD patients awaiting transplant is therefore critical to mitigate sensitization and improve transplant outcomes.^12^ In kidney recipients, preformed anti-HLA antibodies elevate the risk of antibody-mediated rejection, leading to damage to the transplanted organ. These antibodies adhere to the endothelium, causing hyperacute and acute graft rejection, which results in poor survival outcomes.^16^

Reducing the formation of new HLA antibodies and minimizing existing ones in kidney patients can enhance transplant success. Research indicates that HLA class I antibodies correlate with acute rejection,^17^ while HLA class II antibodies are linked to chronic rejection.^18^

### Objective

The aim of this study was to evaluate the influence of the number, volume and type of blood components received on the appearance of anti-HLA antibodies in patients on the waiting list for kidney transplantation, as well as to evaluate the effect of time from the blood transfusion to the detection of the appearance of HLA antibodies.

## Materials and methods

### Study population

A one-year (2016) retrospective study involved 115 cadaveric kidney transplant candidates from South Bačka and Novi Sad, Serbia. Of these, 69 received blood component transfusions for sensitization control, while 46 did not. The study adhered to the Declaration of Helsinki, ensuring respondent anonymity, with data presented in aggregated form. This study was approved by the Ethics Committee of the Faculty of Medicine in Novi Sad and Clinical Center of Vojvodina (Novi Sad) - No. 01-14/17-3.

Demographic data and transfusion history were retrospectively collected from the database of the Vojvodina Blood Transfusion Institute. Sensitization was defined as the presence of positive HLA antibodies or a positive Complement Dependent Cytotoxicity (CDC) test result on at least one occasion. The inclusion criteria were patients on the cadaveric kidney transplant waiting list, undergoing four anti-HLA antibody tests annually (in accordance with the guidelines of the European Federation of Immunogenetics),^19^ and receiving blood component transfusions before sensitization testing.

The blood components that patients received included: packed RBCs, leukoreduced RBCs, resuspended RBCs depleted of WBCs and platelets, resuspended RBCs, fresh frozen plasma, double-leukoreduced RBCs, leukoreduced and washed RBCs, fresh frozen plasma without cryoprecipitate, leukocyte-depleted platelets, and preserved whole blood. For the purposes of this study, we categorized the blood components into two groups: leukodepleted (without WBCs) and those containing WBCs. This classification helps to better understand the impact of the WBC content on sensitization.

### Detection of anti-HLA antibodies

Detection of anti-HLA antibodies was performed in the tissue typing laboratory of the Vojvodina Blood Transfusion Institute. The results of detection of anti-HLA antibodies used in the analysis are based on the application of three methods: CDC test, Enzyme-Linked Immunosorbent Assay (ELISA) and Luminex bead-based technology (LMX).

Serum samples were screened for preformed anti-HLA class I antibodies using the CDC test following Terasaki's method as per National Institutes of Health (NIH) guidelines. Samples (1 µL) were dispensed onto Terasaki trays with a 20-cell panel sourced from diverse HLA donors, covering the Vojvodina population alleles. Controls comprised negative sera from male AB donors and positive pooled sera (>80 % PRA). Fresh donor cells (1 µL of a 2 × 10^6^/mL suspension) were added and incubated at room temperature for 30 min. Subsequently, 5 µL of rabbit complement was added to each well, followed by a 60-minute incubation at 22 °C. Lysed and viable lymphocytes were assessed using 5 % eosin dye and 37 % formaldehyde under an inverse phase contrast microscope. Reactivity against 10 % or more panel members indicated significant presensitization. The performance of the ELISA and LMX tests for detecting target analytes was assessed. Reference samples with known concentrations were prepared and tested using both assays. In the ELISA procedure, microplate wells were initially coated with specific capture molecules, followed by incubation with the reference samples. Subsequent steps involved detection of antibody binding, washing, and the addition of a substrate solution for colorimetric detection. The LMX was conducted using the Luminex 100 System with xMap technologies and xPONENT Software, designed for protocol-based data acquisition and robust data regression analysis. The reagents for the LMX were supplied by Immucor (Norcross, GA, USA). Specifically, the LIFECODES LifeScreen Deluxe (LMX) is a Luminex® Screening Assay designed for the detection of IgG antibodies against HLA Class I and Class II molecules of human origin. The test involved the preparation of microspheres coated with capture molecules, incubating them with reference samples, adding fluorescently labeled detection molecules, and quantifying the resulting fluorescence intensity using a flow cytometer. Data were analyzed using the MATCH IT! Antibody software.

Analyzed parameters included demographic (gender, age), ABO type, RhD type, anti-HLA class I and II antibody tests, PRA percentage, blood component details (number, volume, type), and time since blood transfusion to HLA antibody detection.

### Statistical analysis

Data were processed in the IBM Statistical Package for Social Sciences, version 23 (SPSS Inc., Chicago, IL) and MedCalc for PC (Medcalc Software, Mariakerke, Belgium). Data analysis methods used descriptive and inferential statistics.

Numeric variables were presented as mean (±SD), while discrete outcomes were shown as absolute and relative ( %) frequencies. Two groups were formed based on sensitized patient values, with group comparability assessed through demographic data and follow-up duration. Normality and heteroskedasticity of continuous data were examined using Shapiro-Wilk and Levene’s tests, respectively. Continuous outcomes were compared using unpaired Student *t*-test, Welch *t*-test, or Mann-Whitney U test based on data distribution. Discrete outcomes were compared using chi-squared or Fisher’s exact test accordingly.

Receiver Operating Characteristic (ROC) curves were utilized to predict sensitization based on the number and volume of blood units received. The area under the curve and 95 % confidence intervals were calculated. Multivariate logistic regression was conducted to assess the relationship between sensitization and explanatory variables, including the number of blood components received, number of blood units received, volume of received blood in milliliters, and types of received blood components (with or without WBCs reduction). Data were checked for multicollinearity, heteroskedasticity and normality. Statistical significance was set at a p-value <0.05. Sampling weights were applied during statistical analysis, and results are presented in tables, charts, and diagrams.

## Results

Of 115 patients on the kidney transplant waiting list, 46 (40%) had no history of receiving blood units. Consequently, the studied group included the 69 patients (60%) with a history of receiving blood units before transplantation. Table 1 shows data on the gender and ABO type of the examined patients. A total of 69 patients participated in the study, of which 53.7% were sensitized and 46.3% were not sensitized. Among the sensitized patients, almost 60% were female, with the A, RhD positive blood type predominating. Median age was 56.0 (interquartile range [IQR]: 19.0) in patients who were sensitized and 52.0 (IQR 19.25) in patients who were non-sensitized (Median [Yes - No] = 4.0; p-value = 0.032).

**Table 1:** Demographic and clinical characteristics.

	Sensitized % (53.7 %)	Non-sensitized % (46.3 %)	p-value
Gender Male Female	40.5 59.5	71.9 28.1	0.015
Age	56.0 (IQR: 19.0)	52.0 (IQR: 19.25)	0.032
ABO type A B AB 0	43.2 21.6 16.2 18.9	50 12.5 6.3 31.3	0.318
RhD type positive negative	59.2 10.8	81.2 18.8	0.496

IQR: Interquartile range

A statistically significant difference in the detection of class I and class II HLA antibodies was demonstrated using different techniques (ELISA and LMX) between patients who were sensitized and those who were not. Of the sensitized patients, HLA class I antibodies were detected more often, and in a higher percentage by the LMX technique (81.08%) than by the ELISA technique (45.95%) (Table 2).

**Table 2:** HLA antibodies detection between groups.

Variable	Sensitized % (53.7)	Non-sensitized % (46.3)	p-value
ELISA I not valid detected not detected	8.11 45.95 45.95	12.5 0.0 87.5	<0.001
ELISA II not valid detected not detected	8.11 27.03 64.86	12.5 0.0 87.5	0.002
LMX I not valid detected not detected	0.0 81.08 18.92	3.12 0.0 96.88	<0.001
LMX II not valid detected not detected	0.0 59.46 40.54	3.12 0.0 96.88	<0.001

ELISA I: Investigation of anti-HLA class I antibodies using the ELISA method; ELISA II: Investigation of anti-HLA class II antibodies using the ELISA method; LMX I: Investigation of anti-HLA class I antibodies using the LMX method; LMX II: Investigation of anti-HLA class II antibodies using the LMX method.

**Fig. 1:**
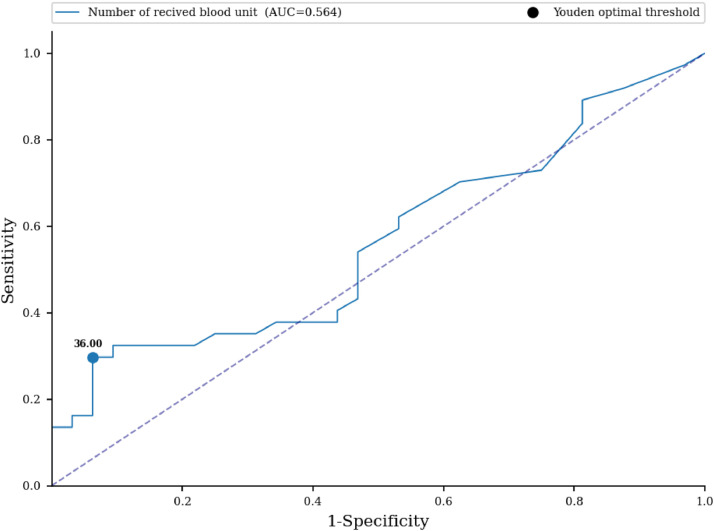
Receiver Operating Characteristic curve - prediction of sensitization in relation to the number of blood units received.

**Fig. 2:**
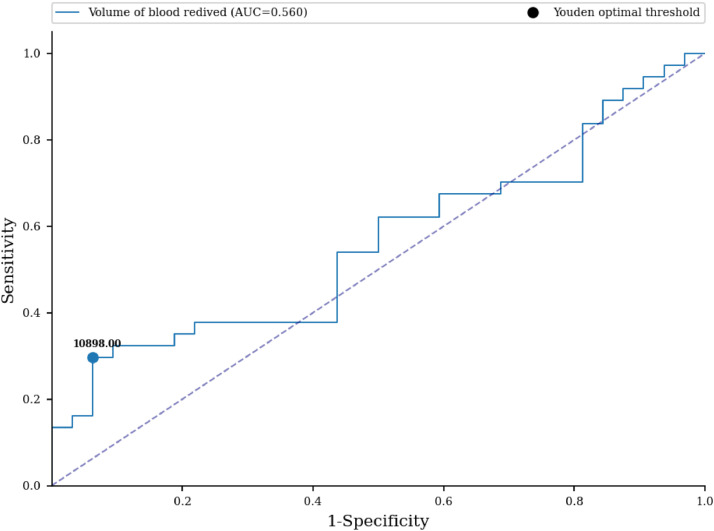
Receiver Operating Characteristic curve - prediction of sensitization in relation to the volume (in milliliters) of blood received.

No statistically significant difference in the number of received blood units was proven between the examined groups (33.27 versus 16.56; p-value = 0.366) (Table 4). An ROC curve (Figure 1) was built to assess the prediction of sensitization in relation to the number of blood units received, with the area under the curve being 0.564 (95% CI: 0.467-0.66) with a cut-off value of 36 units, indicating a weak predictive value of sensitization. Based on the cut off value, we divided the respondents into two groups; a significantly higher percentage of non-sensitized patients received more than 36 units of blood (93.75% versus 70.27%; p-value <0.001) (Table 4). No statistically significant difference was found for the number of blood components received between the two groups (3.19 versus 2.97; p-value = 0.787) (Table 4). No statistically significant difference was identified for the volume of blood received (in milliliters) between the two groups (9.636 L versus 4.673 L; p-value = 0.396) (Table 4). An ROC curve (Figure 2) was performed to assess the prediction of sensitization in relation to volume of blood received with the area under the curve being 0.56 (95% CI: 0.463-0.657) and a cut-off value of 10.898 mL, which shows a weak predictive value of sensitization. Based on the cut off value, we divided the respondents into two groups; a significantly higher percentage of non-sensitized patients received over 11 L of blood (93.75% versus 72.97%; p-value = 0.003) (Table 4). In multivariate analysis, the number of blood components received (OR = 0.88; 95% CI: 0.65-1.21; p-value = 0.437), number of blood unit received (OR = 0.96; 95% CI: 0.77-1.18; p-value = 0.6809), received blood volume in milliliters (OR = 1.0; 95% CI: 1.0-1.0; p-value = 0.5857) were not associated with the rate of sensitization (Table 6).

A significantly higher percentage of sensitization was found in patients who received blood components with WBCs (89.19% versus 68,75%; p-value = 0.006) compared to non-sensitized patients (Table 4). Among patients who received blood components without WBCs, 91.89% were not sensitized and 96.88% were sensitized; however, this difference was not statistically significant (p-value = 0.618) (Table 4). There was a significant difference in the frequency of using blood components containing white blood cells (WBCs) between sensitized and non-sensitized patients (p-value = 0.011); of the sensitized patients, 78.38% received both leukocyte-containing and leukocyte-depleted blood components, whereas 10.81% received only one or the other; in the non-sensitized group, 62.5% received both types of blood components (with and without WBCs), while 31.25% received components without WBCs, and 6.25% received components with WBCs (Table 4). Multivariate analysis showed that the receipt of blood components with and without white blood cells (WBCs) was associated with a higher rate of sensitized patients (OR = 3.62; 95% CI: 1.45–9.04; p-value = 0.0057). Additionally, the receipt of blood components containing WBCs alone was also associated with a higher rate of sensitization (OR = 5.0; 95% CI: 1.17–21.39; p-value = 0.03).

Within six months after transfusion, 21.62% of the patients developed sensitization. An additional 10.81% became sensitized between 6 and 12 months, while in the majority (67.57%) sensitization was detected more than 12 months after the transfusion (Table 4). When analysed according to transfusion-related parameters, the highest sensitization rate was observed in patients who developed HLA antibodies 6-12 months after transfusion (80.0%), compared with 56.82% in those with antibody emergence after more than 12 months, and 40.0% in those with antibodies detected within the first six months (Table 5). Patients who developed HLA antibodies within the first year after transfusion had received a significantly higher number of blood units (p-value <0.001), greater volume of transfused blood (p-value <0.001), and more blood components compared to those with later antibody development (p-value = 0.019). Moreover, the type of transfused blood components differed significantly among groups: patients in the >12-month group predominantly received both leukocyte-containing and leukocyte-reduced components, whereas in the <12-month groups, only leukocyte-reduced components were most frequently administered (p-value <0.001).

According to the Eurotransplant Kidney Allocation System (ETKAS) classification, 51.35% of the group of sensitized patients were transplantable (Group I), 48.65% were immunized (Group II), and there were no highly immunized patients (Group III). No significant difference was demonstrated in the sensitized patients according to the ETKAS classification (p-value = 0.869). In this study, significant differences in HLA antibody detection were found between PRA-positive and PRA-negative groups (Table 3; p-value <0.001). Among PRA-positive patients, 61.11% had antibodies for both HLA class I and II, while 27.78% tested positive for HLA class I antibodies alone, and none had isolated HLA class II antibodies. In contrast, PRA-negative patients had a lower overall rate of sensitization, with 15.69% showing dual sensitization and 7.84% testing positive for isolated HLA class II antibodies. Additionally, PRA-negative patients had a higher percentage of negative HLA results (62.75%) compared to the PRA-positive group (26.09%).

**Table 3:** Human leukocyte antigen antibody detection between panel reactive antibody groups.

	**HLA I**	**HLA I and II**	**HLA II**	**negative**	**Total**	**p-value**
**PRA positive**	5 27.78 % 41.67 % 7.25 %	11 61.11 % 57.89 % 15.94 %	0 0.0 % 0.0 % 0.0 %	2 11.11 % 5.88 % 2.9 %	18 26.09 %	<0.001
**PRA negative**	7 13.73 % 58.33 % 10.14 %	8 15.69 % 42.11 % 11.59 %	4 7.84 % 100.0 % 5.8 %	32 62.75 % 94.12 % 46.38 %	51 73.91 %	
**Total**	12 17.39 %	19 27.54 %	4 5.8 %	34 49.28 %	69 100 %	

PRA: panel reactive antibodies; HLA: human leukocyte antigen.

**Table 4:** Characteristics of blood component in relation to sensitization.

Variable	Sensitized(53.7 %)	Non-sensitized(46.3 %)	p-value
Number of blood unit received	12.0 (IQR: 36.0)	10.5 (IQR: 16.5)	0.198
Number of blood units received (based on cut off) - % 0–36 >36	29.73 70.27	6.25 93.75	<0.001
Number of blood components received – mean (± SD)	3.19 (± 1.79) Range: (1.0–8.0)	2.97 (± 1.47) Range: (1.0–6.0)	0.696
Number of blood components received (based on cut off) 0–6 >6	10.81 89.19	3.12 96.88	0.106
Volume of blood received (mL)	3520 (IQR: 9859)	2992.5(IQR: 3916.75)	0.226
Volume of blood received in liters (based on cut off) - % 0–11 >11	27.03 72.97	6.25 93.75	0.003
Received blood component with WBCs - % Yes No	89.19 10.81	68.75 31.25	0.006
Received blood component without WBCs - % Yes No	91.89 8.11	96.88 3.12	0.618
Received blood component - % with WBCs without WBCs both	10.81 10.81 78.38	6.25 31.25 62.5	0.011

WBC: White blood cell; HLA: human leukocyte antigen; IQR: Interquartile range.

**Table 5:** Time from blood transfusion to HLA antibody detection.

	<6 month % (29.0)	6–12 month % (7.2)	>12 month % *n* = (63.8)	p-value
Gender – % male female	55.0 45.0	80.0 20.0	52.27 47.73	0.248
Age – years	51.85 (± 10.89) Range: (31.0–71.0)	53.8 (± 14.2) Range: (28.0–64.0)	53.5 (± 11.55) Range: (35.0–74.0)	0.642
Sensitization - % Yes No	40.0 60.0	80.0 20.0	56.82 43.18	0.046
Number of blood units received	38.05 (± 43.11) Range: (2.0–167.0)	65.4 (± 74.56) Range: (9.0–188.0)	15.3 (± 19.46) Range: (1.0–107.0)	<0.001
Number of blood components received	3.6 (± 1.91) Range: (1.0–8.0)	3.8 (± 1.4) Range: (2.0–6.0)	2.77 (± 1.45) Range: (1.0–6.0)	0.019
Volume of blood received (mL)	11,054.75 (± 12,543.96) Range: (490.0–47,142.0)	18,263.4 (± 20,259.7) Range: (2765.0–50,097.0)	4401.14 (± 6070.36) Range: (165.0–35,083.0)	<0.001
Received blood component - % with WBCs without WBCs both	0.0 40.0 60.0	0.0 0.0 100.0	13.64 13.64 72.73	<0.001

WBC: Leukocyte; RBC: Red blood cell.

In relation to the type of blood components received, the only significant difference between sensitized and non-sensitized patients was demonstrated when using packed RBCs (89.19% versus 68.75%; p-value = 0.006) (Table 7). The use of other types of blood components is shown in Table 8. In multivariate analysis, blood transfusion (OR = 3.75; 95% CI:1.52-9.26; p-value = 0.0042) were associated with higher rates of sensitization (Table 6).

**Table 6:** Multivariate analysis.

**Characteristic of blood components in relation to sensitization**		
	**Odds Ratio**	**p-value**
Intercept		
	1.2 (0.546–2.64)	0.649
Number of received blood units		
Risk for each 1-unit increase	0.957 (0.775–1.18)	0.681
Number of received blood components		
Risk for each 1-unit increase	0.884 (0.649–1.21)	0.437
Received blood volume in milliliters		
Risk for each 1-unit increase	1 (0.999–1)	0.586
**Type of blood components in relation to sensitization**		
**Intercept**		
	1.45 (0.969–2.17)	0.0706
Reference: blood components with and without WBCs		
blood components containing WBCs	1.38 (0.389–4.89)	0.619
leukodepleted blood components	0.276 (0.111–0.688)	0.005
**The effect of the use of packed RBCs on sensitization**		
**Intercept**		
	0.4 (0.176–0.908)	0.028
**packed RBCs**		
	3.75 (1.52–9.26)	0.004

WBC: Leukocyte; RBC: Red blood cell.

**Table 7:** Transfusion of different blood components between groups.

Variable	Sensitized %(53.7)	Non-sensitized %(46.3)	p-value
Packed RBCs Yes No	89.19 10.81	68.75 31.25	0.006
Leukoreduced RBCs Yes No	40.54 59.46	40.62 59.38	>0.999
Resuspended RBCs depleted of WBCs and platelets Yes No	62.16 37.84	53.12 46.88	0.368
Resuspended RBCs Yes No	62.16 37.84	62.5 37.5	>0.999
Fresh Frozen Plasma Yes No	29.73 70.27	40.62 59.38	0.246
Double Leukoreduced RBCs Yes No	5.41 94.59	3.12 96.88	0.686
Leukoreduced and washed RBCs Yes No	5.41 94.59	3.12 96.88	0.686
Fresh Frozen Plasma without cryoprecipitate Yes No	13.51 86.49	18.75 81.25	0.545
leukocyte-depleted platelets Yes No	2.7 97.3	3.12 96.88	>0.999
preserved whole blood Yes No	8.11 91.89	3.12 96.88	0.285

WBC: Leukocyte; RBC: Red blood cell.

## Discussion

Compared to the findings of this study, where 53.7 % of the subjects were sensitized, other studies reported slightly lower rates. The study of Loupy et al. in 2012^20^ found pre-transplant sensitization in up to 30 % of kidney transplant candidates. Susal et al.^21^ reported nearly 25 % of patients on kidney waiting lists had pretransplant anti-HLA antibodies. Of transfused patients, females showed higher rates of sensitization (33-60 %) compared to males (17-34 %), consistent with the findings of this study with 59.5 % of sensitized women.^22^

Several studies have investigated the relationship between blood group antigens and HLA antibodies with varying results. Rouger et al.^23^ suggested that blood group antigens could influence the detection and formation of HLA antibodies, which is a crucial factor for transplant compatibility and outcomes. Conversely, Erikoglu et al.^24^ found that blood type does not directly impact the distribution of HLA antigens. Supporting this, Cruz-Tapias et al.^25^ reported that while blood group antigens might have indirect effects on HLA antibody formation and detection - potentially complicating test interpretation due to cross-reactivity -they are not directly linked to HLA antibody detection but can influence the overall immune response. In contrast, this study found that ABO blood type did not influence the detection of HLA antibodies, aligning with the observations of Erikoglu et al.^24^ and suggesting that, within this cohort, the blood group antigens do not significantly affect HLA antibody detection.

In the study by Pandey et al. in 2022,^26^ transfused blood showed a high rate of alloimmunization for HLA class I antigens. Picascia et al.^27^ observed a higher frequency of anti-HLA antibodies for class I compared to class II, although not statistically significant. In this study, of the sensitized patients receiving blood transfusions, HLA class I antibodies were more frequently detected by LMX (81.08 %) than ELISA (45.95 %). Of the PRA-positive patients, HLA class I antibodies were detected in the highest percentage (88.89 %), and HLA class II antibodies in 61.11 %. Vasic et al.^28^ found that nearly half of the patients received less than ten units of blood, with an average sensitization level of 13.61 %. Handa et al.^29^ reported no significant association between the number of transfused units and alloimmunization. Similarly, Vasic et al.^28^ noted higher sensitization levels in patients receiving more than 3000 mL of transfused blood. In the present study, patients receiving less than 11 L of blood components had an average sensitization level (PRA) of 4.82 %, compared to 25 % in those receiving more.

Bilgin et al.^30^ found that transfusion of leukocyte-depleted platelets significantly reduces the formation of anti-HLA antibodies. In the current study, blood components with depleted WBCs showed a higher rate of sensitized patients (12.5 %) compared to those without (5 %), but the difference was not statistically significant (p-value = 0.03). Vasic et al.^28^ similarly found no significant difference in sensitization levels between patients receiving leukoreduced RBC units and those who did not. Previous studies by Karpinski et al.^31^ also showed no difference in allosensitization rates between standard and leukoreduced RBC transfusions. Despite WBC reduction, alloimmunization rates vary widely, ranging from 7–44 % among recipients of leukocyte-reduced blood transfusions and from 20–50 % among control recipients of non-leukoreduced blood components.^32^ As explained in one study,^33^ sensitization levels are similar between recipients of leukoreduced and non-leukoreduced blood components. The authors suggest that residual WBCs and RBCs carrying HLA antigens may contribute to the reduced effectiveness of leukoreduced units in preventing sensitization. Vasić et al.^28^ observed lower sensitization levels with longer periods since the last transfusion, suggesting that the longer the time elapsed, the less likely it is for the patient to develop alloimmunization. This contrasts with the findings of the present study, where patients who developed HLA antibodies more than 12 months post-transfusion still showed a relatively high rate of sensitization (56.82 %). However, a closer examination of these data reveals that those who developed antibodies earlier (within 6–12 months) exhibited the highest sensitization rates (80 %), indicating that the intensity and frequency of transfusions within the first year play a critical role in sensitization. While the findings of Vasić et al. highlight the potential decline in sensitization over time, the results of this study emphasize the significance of transfusion-related parameters, such as the number of transfusions and blood components, in the development of HLA antibodies, which could suggest that the type and extent of exposure to transfused blood have a more substantial impact on the sensitization process than the mere passage of time.

In the study of Vasic et al. in 2013,^28^ the mean PRA value in patients receiving blood components was 16.04 %, consistent with the average PRA value of the current study with 15.54 %, both falling into the ETKAS II group (PRA 6–85 %). Karahan et al.^34^ reported that among patients with positive PRA, 47.6 % had positive HLA class I antibodies, 16.7 % had positive HLA class II antibodies, and 35.7 % had positive HLA antibodies for both classes. In this study, 27.78 % of the PRA-positive patients had HLA class I, 61.11 % had HLA class I and II, and none had only class II. Additionally, 11.11 % of PRA-positive patients were negative for HLA antibodies. The findings of this study show a higher prevalence of dual sensitization (both class I and II) compared to Karahan et al.^34^ Marfo K. et al.^16^ reported 35 % of patients on the waiting list had PRA values >0 %, with 15 % highly sensitized (PRA levels >80). In the present study, according to the ETKAS classification, 51.35 % of the sensitized patients were transplantable (Group I, PRA<6 %), 48.65 % were immunized (Group II, PRA 6–85 %), and none were highly immunized (Group III, PRA >85 %). No significant difference was found in sensitized patients based on the ETKAS classification. In this study, a significant difference in sensitization rates was found between patients who received packed RBCs and those who did not, with a 3.75 times higher likelihood of sensitization in the former group. Recent analysis using LMX technology from the US Renal Data System revealed that RBC transfusions can strengthen and broaden HLA antibodies. Laffell et al.^10^ reported a 20 % antibody response rate in patients receiving RBCs transfusions, leading to a tenfold increased relative risk of broad sensitization and a 32-point mean increase in PRA. These findings suggest a causal link between RBCs transfusions and clinically relevant HLA antibody development, resulting in a significant decrease in available donor organs. Therefore, minimizing transfusions whenever possible for patients on the transplant waiting list is crucial.

## Conclusion

The study findings suggest that while the total number, volume, and units of blood components received do not significantly contribute to an increase in anti-HLA antibodies or sensitization, the kind of blood component plays a crucial role. Specifically, transfusions involving blood components containing leukocytes are more likely to lead to sensitization. Among blood cell components, the transfusion of packed RBCs is associated with a higher incidence of sensitization compared to other blood components. Additionally, the time elapsed since transfusion is a significant factor, with a longer interval post-transfusion being correlated with a higher likelihood of detecting sensitization.

## Funding

This research did not receive any specific grant from funding agencies in the public, commercial, or not-for-profit sectors.

## Conflicts of interest

None.
